# Impact of Technology Use on Behavior and Sleep Scores in Preschool Children in Saudi Arabia

**DOI:** 10.3389/fpsyt.2021.649095

**Published:** 2021-05-21

**Authors:** Doaa Almuaigel, Abrar Alanazi, Mohammed Almuaigel, Foziah Alshamrani, Mona AlSheikh, Nora Almuhana, Mohammad Zeeshan, Mohammed Alshurem, Alaa Alshammari, Kamel Mansi

**Affiliations:** ^1^Special Education Department, College of Education, Imam Mohammed Bin Saud Islamic University, Riyadh, Saudi Arabia; ^2^Neurology Department, King Fahad University Hospital, Al Khobar, Saudi Arabia; ^3^Neurology Department, College of Medicine, Imam Abdulrahman Bin Faisal University, Dammam, Saudi Arabia; ^4^Physiology Department, College of Medicine, Imam Abdulrahman Bin Faisal University, Dammam, Saudi Arabia; ^5^Psychiatry Department, King Fahad University Hospital, Al Khobar, Saudi Arabia; ^6^Psychiatry Department, College of Medicine, Imam Abdulrahman Bin Faisal University, Dammam, Saudi Arabia; ^7^School of Management, University of Bristol, Bristol, United Kingdom

**Keywords:** children, digital technology, impact, parents, perception

## Abstract

**Background:** Pre-school children use digital devices both at home and in kindergarten for communication. However, such technologies can also be used for creativity learning and entertainment. Technology usage might exert a negative impact on the psychosocial development of pre-school children, thus necessitating parental monitoring. Previous studies have recommended early intervention for pre-school children by decreasing the duration of digital devices, spending more time with the family, and participation in motor activities to avoid the ill effects of technology.

**Aim:** To investigate the impact of digital device use on the behavioral and sleep scores of preschool children as perceived by parents in Saudi Arabia (SA).

**Method:** This cross-sectional study was conducted across two regions in SA. It was ethically approved by the ethical review board of Imam Abdulrahman Bin Faisal University. The participants were randomly selected from well-baby hospital records, surveyed and interviewed to obtain data for the following measures: demographic data, technology usage, sleep disturbance scale, and behavior scale. Children with special needs or comorbidities were excluded from the study. Descriptive and multivariate regression analysis were done.

**Results:** We recruited 288 children. Most did not attend schools (63.2%), 22.6% were in kindergarten, and 14.2% were in nursery schools. Smart phones were the most commonly used device by the children (42.4%). Most used the technology for 2–3 h/days (34%). Cartoons were the most commonly sought content (42%). The behavior scores for children aged 18–36 months showed a mean value of 5.1, 3.7, and 4.6 for surgency, negative affect, and effortful control, respectively. Children aged 3–5 years showed a mean value of 4.3, 4, and 4.7 for surgency, negative affect, and effortful control, respectively. Sleep disturbance scores for all children showed a mean value of 12.4, 3.5, 3.8, 8, 7.3, and 2.7 on disorders of initiating and maintaining sleep, sleep-breathing disorders, disorders of arousal, sleep-wake transition disorders, disorders of excessive somnolence, and sleep hyperhidrosis, respectively. The mean total sleep score was 37. Multivariate regression analysis showed significant positive relationship between surgency and three factors namely family income of 10,000–15,000 SR (t = 1.924, *p* = 0.045), fathers' bachelor's degrees (t = 2.416, *p* = 0.16), and owning a video game device (t = 2.826, *p* = 0.005<0.05). Negative affect was significantly associated with fathers' diploma level of education (t = 2.042, *p* = 0.042). Negative significant relationship between effortful control and fathers' secondary level of education (t = −2.053, *p* = 0.041). There was a significant negative relationship between effortful control and owning a TV and video game device (t = −2.35, −2.855, *p* = 0.043, 0.005<0.05, respectively). A significant positive relationship was found between child's sleeping score (worse sleep) and watching technology between 3 and 5 h (t = 2.01, *p* = 0.045), and mothers' unemployment status (t = 2.468, *p* = 0.014).

**Conclusion:** In conclusion, technology use is associated with a negative impact on children sleep and behavior. Owning a digital device, using tablets, screen viewing for more than 3–5 h, and watching movies were significantly associated with negative child's behavior and sleep.

## Introduction

In contemporary society, pre-school children use digital devices both at home and in kindergarten. They use technology not only for communication but also for learning and entertainment. The experience with pre-school children using digital devices is relatively new. Therefore, there is little evidence for its impact on children, sleep, behavior, and psychosocial development. Children primarily use technology for entertainment and curiosity satisfaction; caregivers pay scant attention to the influence of this practice (1).

The aforementioned situation is similar in Saudi Arabia (SA), where the use of communication or electronic entertainment devices is particularly common in the pediatric population (2). Technology usage might impose a negative influence on child's psychosocial development. Therefore, parental awareness and monitoring is necessary (3).

The ill effect of electronic device use on sleep of children can be due to the hormones provoked by excitement, suspense, drama, and conflict that is involved in the media and games that the child is exposed to (4). The secretion of these stress and arousal hormones will result in disruption of sleep quality. The exposure to light emitted from electronic devices can also suppress the secretion of melatonin, a sleep hormone which interferes with the physiologic circadian rhythm (4, 5).

Physical activities will distract the child from screen viewing and is expected to allow restoration of melatonin levels and sleep quality. As afore-mentioned, sleep is very vital for child's health, immune system, and normal mental, physical, and emotional development. Having a normal family atmosphere encourages communication between the child and his parents or sibs and is expected to have a positive effect on the behavior, sleep, and child's speech and language development (6).

The ill effect of technology use on sleep and behavior is not new, but quantifying it at different stages of childhood, from a parental point of view, in the parents' native language, by interview-reinforced-surveys, is this study's contribution.

Our aim is to study the association of screen time viewing on behavior and sleep scores of preschool children in SA. Other demographic factors associations were evaluated to control for their confounding effect.

## Materials and Methods

This is a cross-sectional study that was conducted across two regions in SA. It received ethical approval from the ethical review board of Imam Abdulrahman Bin Faisal University.

### Participants

The study included Saudi children across two regions in SA, namely the Eastern and Central Provinces. We interviewed parents of children aged 1.5–5 years who used technology for any purpose. We excluded children with special needs and comorbidities like chronic illness, congenital syndrome, or disease and parents who could not read or respond to electronic questionnaires.

### Study Procedure

The study participants were randomly selected and recruited from well-baby hospital records and by posting fliers on different social media websites. The fliers requested interested parents of pre-school children (kindergarten, nursery schools, and those at home) to fill out their contact details, including their name, mobile number, e-mail address, city, and if their child had any medical condition. We contacted the parents to decide the best time for the interview. Telephonic interviews were conducted in Arabic by trained and qualified graduate students. Consent was taken verbally at the beginning of the interview and electronically at the beginning of the survey.

### Measures

Our study used a questionnaire that comprised four major sections: demographic and clinical data, technology usage, sleep disturbance score, and preschool behavior rating scale.

### Demographic and Clinical Data

The demographic and clinical data comprised two parts. While the first part consisted of child-related questions, the second part consisted of family-related questions.

### Technology Use

Questions related to the use of technology by a child focused on the duration, type of device, supervision, and content.

### Sleep Disturbance Scale for Children

The Sleep Disturbance Scale for Children (SDSC) is a 26-item questionnaire (7, 8) (Table 1). It consists of the following six subscales: disorders of initiating and maintaining sleep (DIMS), sleep-breathing disorders (SBD), disorders of arousal (DA), sleep-wake transition disorders (SWTD), disorders of excessive somnolence (DOES), and sleep hyperhidrosis (SHY). The questionnaire comprises a five-point Likert scale to evaluate the aforementioned components of sleep disturbance (never, occasionally, sometimes, often, and always). The higher the score, the worser the sleep.

**Table 1:** Five-point likert scale results for the sleep disturbance scale for children.

**Never**	**Occasionally (once or twice per month or less)**	**Sometimes (once or twice per week)**	**Often (thrice or five times per week)**	**Always (daily)**
1	2	3	4	5

### Children's Behavior Questionnaire

The Children's Behavior Questionnaire (CBQ) is presented in two forms. The first form is for children aged 3–7 years (9). The second form is for children aged 18–36 months (10). Both forms include 36 questions and are evaluated at seven levels, ranging from never [1] to always [7] (Table 2). The higher the score, the worse the behavior.

**Table 2:** Response range for the children's behavior questionnaire.

**Never**	**Very rarely**	**Less than half the time**	**About half the time**	**More than half the time**	**Almost always**	**Always**	**Does not apply**
1	2	3	4	5	6	7	8

### Validation and Reliability

The Arabic version of the CBQ and SDSC surveys was used in this study (8). The original translation and validation were done by Abu Khadra and the process involved “translation and back translation. The questionnaire was translated into Arabic by an independent translator and revised by the author. Phrases difficult for parents to understand were changed and if no suitable synonym was available, a verbal explanation was added. The questionnaire was back-translated by a second independent translator. Cronbach's alphas using data from these children were 0.77 for the entire scale, and 0.50 to 0.73 for subscales.”

We assessed the validity and reliability of the Arabic version of the SDSC and CBQ. The method of validation involved a three-step process.

Step 1: the questionnaires were reviewed by two child psychology experts.

Step 2: The questionnaires were piloted on 10 participants.

Step 3: We conducted a principal component analysis to check for their internal consistency. The surveys were eventually revised. The reliability (Cronbach's alpha coefficient) was found to be = 0.71 and = 0.78 for SDSC and CBQ, respectively.

### Data Management

A specific code was assigned to each participant for the baseline data and backup. The codes were secured with a password. A large number of medical graduates were trained to act as data collectors and perform the interviews.

### Statistical Analysis

Descriptive statistics for the nominal variables are presented as frequency and percentage. In contrast, those for the continuous variables are presented as mean and standard deviation. Depending on the normality of the data, the impact of different factors on the sleep and behavior scores were compared using the independent Student's *t*-test and one-way ANOVA or Kruskal-Wallis test for two or more than two groups, respectively.

Multivariate regression analysis was performed to study the strength of association of the independent variables and the sleep and behavior scores. The study also used univariate analysis of variance to examine the relationship between children's duration of time of using technology and their parents' demographics and socioeconomic status. This method was used because the use of duration time is a categorical variable.

IBM SPSS 26 for Windows was used for the analysis. A *P* <0.05 was considered statistically significant.

## Results

Despite the enrolment of 478 children, only 288 children met the inclusion criteria. While there were 71 (24.7%) children aged between 18 and 36 months, 217 (75.3%) children were aged between 3 and 5 years. The sample was balanced for gender, with 145 (50.3%) boys and 143 (49.7%) girls. There were 180 (62.5%) children from the Eastern region, compared to 108 (37.5%) from the Central region. In relation to pre-schooling, 65 (22.6%), 41 (14.2%), and 182 (63.2%) children were from kindergarten, nursery school, and no-school, respectively. The mean birth order of the child in their family was 2.6. The mean number of siblings was 2.7. The marital status of the family was 261 (90.6%) married, 25 (8.6%) divorced, and two (0.7%) widowed. Equal number of houses were owned and rented (*n* = 144). As far as family income, 36 (12.5%), 87 (30.8%), 80 (27.8%), and 85 (29.5%) children belonged to the socioeconomic status <5,000 SR, 5,000 SR−10,000 SR, 10,000 SR−15,000 SR, and >15,000 SR, respectively. The highest education of the father was bachelor's degree in 129 (44.8%) children. Furthermore, 262 (91%) of them were employed or students. Similarly, the highest education of the mothers was bachelor's degree in 177 (61.5%) children. Around 98 (34%) of children mothers were employed or studying. The mean duration of studying/working was 42.6 h/weeks for fathers, compared to 37.7 h/weeks for mothers. Supplementary Table 1 summarizes the characteristics of the study participants.

Multiple devices were used by children. Smart phones were most commonly used by 122 (42.3%) children. The television, tablet, and video games were used by 112 (38.8%), 39 (13.5%), and 15 (5.2%) children, respectively. However, the time of using technology was different each day. Accordingly, 76 (26.4%), 98 (34%), 66 (22.9%), and 48 (16.7%) children used technology for ≤ 1 h, 2–3 h, 3–5 h, and >5 h, respectively. While 159 (55.2%) children preferred to spend their leisure time with their family, 89 (30.9%) children preferred using technology. The rest, 40 (13.9%) children preferred to spend their time with their friends. Most children (*n* = 213, 74%) used technology in the presence of their families. Approximately 185 (64.2%) children had permanent access to the internet without any restrictions, compared to 103 (35.8%) children with restricted access. A large proportion of children (*n* = 187, 64.9%) did not have their own device. While 226 (78.5%) parents reported setting technology use limits for their children, 268 (93.1%) parents directly supervised the use of technology by their child. The most common content accessed by children was cartoons, followed by songs, games, and education by 121 (42%), 97 (33.7%), 37 (12.8%), and 33 (11.5%) children, respectively. Supplementary Table 2 outlines the questions on technology use by children.

### Behavior Scores

The behavior scores for children aged 18–36 months showed a mean value of 5.1, 3.7, and 4.6 for surgency, negative affect, and effortful control, respectively (Table 3). In contrast, the scores for children aged 3–5 years showed a mean value of 4.3, 4, and 4.7 for surgency, negative affect, and effortful control, respectively (Table 4).

**Table 3:** Behavior scores for children aged 18–36 months.

	***N***	**Minimum**	**Maximum**	**Mean**	**Standard deviation**
Surgency	71	2.0	6.8	5.1	1.0
Negative affect	71	1.0	5.3	3.7	0.8
Effortful control	71	2.9	6.7	4.6	0.8

**Table 4:** Behavior scores for children aged 3–5 years.

	***N***	**Minimum**	**Maximum**	**Mean**	**Standard deviation**
Surgency	217	1.6	7.0	4.3	0.9
Negative affect	217	1.3	6.4	4.0	1.1
Effortful control	217	1.0	7.0	4.7	1.7

#### Behavior and Technology

There were a significant difference between the effortful control (*P* < 0.001) scores for the type of device most commonly used. In contrast, smartphone users (mea*n* = 4.2 ± 1.6) had a lower effortful control score than television (mea*n* = 5.0 ± 1.2) or tablet users (mea*n* = 5.2 ± 1.7) (Supplementary Table 3). There was a significant difference between the duration of device use in terms of negative affect (*P* = 0.009). Children who used the devices for ≤ 1 h (mea*n* = 3.7 ± 1.0) had a lower negative affect score than those who used them for 3–5 h (mea*n* = 4.2 ± 1.0) (Supplementary Table 4). In terms of surgency (*P* = 0.033) and effortful control (*P* = 0.002), we observed a statistically significant difference between the children owned their device and those who did not. Children who owned the device had lower surgency scores (mea*n* = 4.3 ± 0.9) than those who did not (mea*n* = 4.6 ± 1.0), and higher effortful control scores (mea*n* = 5.1 ± 1.5) than those who did not (mea*n* = 4.5 ± 1.5). There was a significant difference between the most commonly used content and effortful control scores (*P* = 0.023). Children who commonly watched movies (mea*n* = 4.5 ± 1.6) had a lower effortful control score than those who watched games/cartoons (mea*n* = 5.3 ± 1.2) (Supplementary Table 6).

#### Behavior and Demographic

Working or studying hours demonstrated a weak negative correlation with the effortful control score (r = 0.4) for the father.

#### Multivariate Analysis

The results demonstrate significant relationships between surgency and families with income 10,000–15,000 SR (t = 1.924, *p* = 0.045). The results also indicate significant correlation between surgency and fathers' bachelor's degrees (t = 2.416, *p* = 0.16) (Table 5).

**Table 5:** Regression coefficient table showing the strength of association between technology and demography variables and “Surgency.”

**Coefficients**^**a**^						
**Dependent variable: Surgency**						
**Model**		**Unstandardized coefficients**		**Standardized coefficients**	**t**	**Sig**.
		**B**	**Std. Error**	**Beta**		
1	(Constant)	4.821	1.072		4.499	0.000
	<5,000	0.277	0.220	0.092	1.261	0.209
	**10,000–15,000**	**0.312**	**0.162**	**0.140**	**1.924**	**0.045**
	15,000+	0.177	0.180	0.081	0.986	0.325
	Father secondary level of education	0.117	0.169	0.051	0.692	0.490
	Father diploma level of education	0.033	0.179	0.012	0.185	0.853
	**Father bachelor level of education**	**0.458**	**0.190**	**0.164**	**2.416**	**0.016**
	Mother secondary level of education	0.042	0.168	0.017	0.248	0.804
	Mother diploma level of education	0.348	0.228	0.096	1.530	0.127
	Mother postgraduate level of education	−0.093	0.243	−0.026	−0.383	0.702
	Father unemployment status	−0.133	0.229	−0.038	−0.583	0.561
	Mother unemployment status	−0.007	0.072	−0.007	−0.095	0.924
	Most common used smart phone	−1.248	1.031	−0.618	−1.211	0.227
	Most common used tablet	−0.409	0.522	−0.281	−0.783	0.434
	Most common used TV	−1.098	1.031	−0.537	−1.065	0.288
	Most common used video games	−1.375	1.064	−0.297	−1.293	0.197
	One hour and less	0.134	0.156	0.059	0.863	0.389
	3–5 h	0.190	0.165	0.080	1.151	0.251
	5+ h	0.139	0.185	0.052	0.751	0.453
	Own device tablet	0.407	0.260	0.126	1.565	0.119
	Own device TV	0.430	0.353	0.090	1.216	0.225
	**Own device video games**	**0.516**	**0.183**	**0.247**	**2.826**	**0.005**

a *Dependent Variable: Surgency. Bold values indicates significant relationship*.

In term of negative affect of behavior, the regression results demonstrate only a significant relationship with fathers' diploma level of education (t = 2.042, *p* = 0.042) (Table 6).

**Table 6:** Regression coefficient table showing the strength of association between technology and demography variables and “Negative affect.”

**Coefficients**^**a**^						
**DV: Dependent Variable: Negative_Affect**						
**Model**		**Unstandardized coefficients**		**Standardized coefficients**	**t**	**Sig**.
		**B**	**Std. Error**	**Beta**		
1	(Constant)	2.627	1.099		2.392	0.017
	<5,000	0.135	0.226	0.043	0.596	0.552
	10,000–15,000	0.203	0.166	0.089	1.223	0.222
	15,000+	−0.031	0.184	−0.014	−0.170	0.865
	Father secondary level of education	0.044	0.174	0.018	0.251	0.802
	**Father diploma level of education**	**0.374**	**0.183**	**0.131**	**2.042**	**0.042**
	Father bachelor level of education	0.209	0.194	0.073	1.077	0.282
	Mother secondary level of education	−0.198	0.172	−0.079	−1.154	0.250
	Mother diploma level of education	0.247	0.233	0.066	1.057	0.291
	Mother postgraduate level of education	−0.112	0.249	−0.031	−0.449	0.654
	Father unemployment status	−0.090	0.235	−0.025	−0.382	0.703
	Mother unemployment status	0.112	0.074	0.103	1.504	0.134
	Most common used smart phone	1.072	1.056	0.516	1.015	0.311
	Most common used tablet	0.585	0.536	0.390	1.092	0.276
	Most common used TV	1.127	1.057	0.535	1.066	0.287
	Most common used video games	0.896	1.090	0.188	0.822	0.412
	One hour and less	−0.239	0.160	−0.103	−1.497	0.136
	3–5 h	0.323	0.169	0.132	1.908	0.057
	5+ h	−0.147	0.189	−0.053	−0.776	0.438
	Own device tablet	−0.160	0.266	−0.048	−0.601	0.548
	Own device TV	0.455	0.362	0.092	1.257	0.210
	Own device video games	−0.098	0.187	−0.045	−0.523	0.602

a *Dependent Variable: Negative_Affect. Bold values indicates significant relationship*.

As for effortful control, the regression results show a negative significant relationship between effortful control and fathers' secondary level of education (t = −2.053, *p* = 0.041). Likewise, the results demonstrate negative significant relationships between effort control and TV and video games as owning devices (t = −2.35, −2.855, *p* = 0.043, 0.005<0.05 respectively) (Table 7).

**Table 7:** Regression coefficient table showing the strength of association between technology and demography variables and “Effortful control.”

**Coefficients**^**a**^						
**Dependent variable: Effortful_Control**						
**Model**		**Unstandardized coefficients**		**Standardized coefficients**	**t**	**Sig**.
		**B**	**Std. Error**	**Beta**		
1	(Constant)	5.722	1.589		3.601	0.000
	<5,000	−0.210	0.326	−0.045	−0.643	0.521
	10,000–15,000	0.108	0.240	0.031	0.448	0.655
	15,000+	−0.193	0.267	−0.057	−0.724	0.470
	**Father secondary level of education**	**-0.515**	**0.251**	**-0.146**	**-2.053**	**0.041**
	Father diploma level of education	0.123	0.265	0.029	0.465	0.642
	Father bachelor level of education	0.188	0.281	0.044	0.667	0.505
	Mother secondary level of education	0.135	0.249	0.036	0.541	0.589
	Mother diploma level of education	−0.066	0.337	−0.012	−0.196	0.845
	Mother postgraduate level of education	0.458	0.360	0.084	1.272	0.205
	Father unemployment status	0.424	0.339	0.079	1.249	0.213
	Mother unemployment status	−0.109	0.107	−0.067	−1.016	0.311
	Most common used smart phone	−0.808	1.528	−0.261	−0.529	0.597
	Most common used tablet	−0.119	0.775	−0.053	−0.154	0.878
	Most common used TV	−0.037	1.529	−0.012	−0.024	0.981
	Most common used video games	−0.238	1.577	−0.033	−0.151	0.880
	One hour and less	0.277	0.231	0.080	1.197	0.232
	3–5 h	0.160	0.245	0.044	0.654	0.514
	5+ h	−0.023	0.274	−0.006	−0.084	0.933
	Own device tablet	−0.222	0.385	−0.045	−0.577	0.564
	**Own device TV**	**-1.066**	**0.524**	**-0.144**	**-2.035**	**0.043**
	**Own device video games**	**-0.773**	**0.271**	**-0.241**	**-2.855**	**0.005**

a *Dependent Variable: Effortful_Control. Bold values indicates significant relationship*.

### Sleep Scores

The sleep disturbance scores (SDSC) for all children showed a mean value of 12.4, 3.5, 3.8, 8, 7.3, and 2.7 on DIMS, SBD, DA, SWTD, DOES, and SHY, respectively. In addition, the mean total score was 37.3 (Table 8).

**Table 8:** Sleep disturbance scores for all children.

	***N***	**Minimum**	**Maximum**	**Mean**	**Standard deviation**
DIMS	288	7	30	12.4	4.5
SBD	288	3	15	3.5	1.6
DA	288	3	14	3.8	1.6
SWTD	288	6	25	8.0	2.8
DOES	288	5	23	7.3	3.2
SHY	288	2	10	2.7	1.7
TOTAL	288	26	100	37.7	9.9

*DIMS, Disorders of Initiating and Maintaining Sleep; SBD, Sleep-Breathing Disorders; DA, Disorders of Arousal; SWTD, Sleep-Wake Transition Disorders; DOES, Disorders of Excessive Somnolence; SHY, Sleep Hyperhidrosis*.

#### Sleep and Technology

The results of the study demonstrated significant differences between different durations of screen-time use (<1 h, 2–3 h, 3–5 h, and >5 h and DOES (*P* < 0.001). Likewise, the results showed significant differences between screen-time viewing duration and SHY (*P* < 0.001). For example, children who used the devices for ≤ 1 h (mean = 3.7 ± 1.0) had a higher SHY score than those who used them for 2–3 h (mea*n* = 2.4 ± 1.2). The results of the study also indicated a significant difference between the most commonly used content (movies, songs, educational, and games/cartons) and SBD (*P* = 0.024) as well as SHY (*P* = 0.007). For instance, children who almost commonly watched movies (mea*n* = 3.8 ± 2.3) had a higher SBD score than those who watched games/cartoons (mea*n* = 3.0 ± 0.2). Likewise, children who commonly watched educational content (mea*n* = 3.5 ± 2.9) had a higher SHY score than those who watched games/cartoons (mea*n* = 2.2 ± 0.7).

#### Sleep and Demographic

We observed a weak positive correlation between the employment or studying hours per week and the DIMS score (r = 0.21) for the father. There was a higher positive correlation with the employment or studying hours per week for the mother, leading to a higher DIMS score. Children whose mothers were employed or studying had lower DIMS scores (mean = 11.5 ± 3.7, *P* = 0.011), compared to those whose mothers were not (mean = 12.8 ± 4.8). In addition, children with employed or student mothers had lower DA (mean = 3.5 ± 1.0, *P* = 0.010), SHY (mean = 2.4 ± 1.3, *P* = 0.019), and total scores (mean = 35.4±7.9, *P* = 0.002) than those whose mothers did not (Table 9). Children belonging to families with a monthly income of 5,000–10,000 SR (mean = 8.1 ± 3.9) had a higher DOES score than those with a monthly income of 10,000–15,000 SR (mean = 6.7 ± 2.2). Likewise, children belonging to families with a monthly income <5,000 SR (mean = 4 ± 3.0) had a higher SHY score than all other groups (Supplementary Table 5).

**Table 9:** Regression coefficient table showing the strength of association between technology and demography variables and “Sleep Disturbance Scale for Children” scores.

**Coefficients**^**a**^						
**Dependent variable: Sleep**						
**Model**		**Unstandardized coefficients**		**Standardized coefficients**	**t**	**Sig**.
		**B**	**Std. Error**	**Beta**		
1	(Constant)	47.762	8.242		5.795	0.000
	<5,000	0.065	1.714	0.003	0.038	0.970
	10,000–15,000	−1.363	1.248	−0.080	−1.092	0.276
	15,000+	−0.011	1.391	−0.001	−0.008	0.994
	Father secondary level of education	−0.815	1.304	−0.046	−0.625	0.532
	Father diploma level of education	1.480	1.358	0.070	1.090	0.277
	Father bachelor level of education	0.587	1.484	0.027	0.396	0.693
	Mother diploma level of education	2.217	1.972	0.079	1.124	0.262
	Mother bachelor level of education	0.019	1.290	0.001	0.015	0.988
	Mother postgraduate level of education	−0.393	2.190	−0.014	−0.179	0.858
	Father unemployment status	1.062	1.759	0.039	0.604	0.547
	**Mother unemployment status**	**1.364**	**0.553**	**0.169**	**2.468**	**0.014**
	Most common used smart phone	−10.381	7.840	−0.666	−1.324	0.187
	**Most common used tablet**	**-7.594**	**3.972**	**-0.682**	**-1.912**	**0.047**
	Most common used TV	−12.443	7.842	−0.790	−1.587	0.114
	**Most common used video games**	**-15.730**	**8.086**	**-0.445**	**-1.945**	**0.043**
	One hour and less	−0.044	1.201	−0.003	−0.037	0.970
	**3–5 h**	**2.540**	**1.263**	**0.140**	**2.010**	**0.045**
	5+ h	1.537	1.423	0.074	1.080	0.281
	**Own device tablet**	**-4.608**	**2.004**	**-0.182**	**-2.299**	**0.022**
	Own device TV	−0.285	2.695	−0.008	−0.106	0.916
	Own device video games	−1.629	1.391	−0.101	−1.172	0.242

a *Dependent Variable: Sleep1. Bold values indicates significant relationship*.

#### Multivariate Analysis

The regression coefficients of this study present the results of relationships between sleeping patterns of pre-school children and their families' background characteristics. The results demonstrate significant relationship between watching technology between 3 and 5 h a day and higher SDSC or sleep disruption. Owning a device (t = −2.299, *p* = 0.022<0.05) and using tablets (t = −1.912, *p* = 0.047) were associated with higher SDSC scores or more sleep disturbance (t = −1.912, *p* = 0.047). Furthermore, there was significant positive relationship between children's SDSC and mothers' unemployment status (t = 2.468, *p* = 0.014) (Table 9).

### Univariate Analysis of Variance

The results of univariate analysis demonstrate a significant association between the duration of using technology (screen time viewing) and fathers' socioeconomic status (F = 0.192, *p* = 0.046 <0.05). Another significant finding was the interaction between mothers' highest education level and mothers' employment status which had a significant relationship (F = 3.374, *P* = 0.019 <0.05) with the duration of children's use of technology (Table 10).

**Table 10:** Relationship between duration of using technology and children's family demographics and socioeconomic status.

**Coefficients**					
**Dependent variable: Use of technology in hours**					
**Source**	**Type III Sum of Squares**	**df**	**Mean Square**	**F**	**Sig**.
Corrected Model	97.969*a*	78	1.256	1.248	0.110
Intercept	287.904	1	287.904	286.056	0.000
Socioeconomic	3.383	3	1.128	1.121	0.342
Father highest education level	3.654	3	1.218	1.210	0.307
Mother highest education level	1.461	3	0.487	0.484	0.694
Father employment status	0.169	1	0.169	0.168	0.683
Mother employment status	0.482	1	0.482	0.479	0.490
Socioeconomic of father (income)	14.588	8	1.923	1.912	0.046
Socioeconomic of mother (income)	13.695	8	1.712	1.701	0.100
Socioeconomic ^*^ father employment (interaction)	3.555	2	1.777	1.766	0.174
Socioeconomic ^*^ mother employment (interaction)	1.613	3	0.538	0.534	0.659
Father highest education for ^*^ mother highest education (interaction)	4.683	9	0.520	0.517	0.861
Father highest education^*^ father employment (interaction)	5.352	3	1.784	1.772	0.153
Father highest education ^*^ mother employment (interaction)	1.227	3	0.409	0.406	0.749
Mother highest education^*^ father employment (interaction)	0.837	1	0.837	0.832	0.363
Mother highest education ^*^ mother employment (interaction)	10.186	3	3.395	3.374	0.019
Corrected total	308.319	287			

* *Means interaction*.

Since there were several comparisons, it has become imperative to use a correction method such as Bonferroni test. The Bonferroni test is used when we have many dependent and independent tests and are performed simultaneously. While the level of significance (α) may be adequate for every individual comparison, but it is not suitable for multiple comparisons, therefore, we use Bonferroni correction method. The *p*–adjust (*p*–values) (1), 1.000, 1.000, 1.000, 1.000, 0.314, 1.000, 0.314, 1.000, 1.000, 1.000, 1.000. These Bonferroni results indicate that none of the educational attainment (factor) remain significant in affecting children's sleeping.

## Discussion

Our results showed significant negative impact of screen viewing time on child sleep quality and behavior indicators. The study demonstrated a significant relationship between screen viewing time and father's socioeconomic status. Furthermore, the study found a significant interaction between mother's highest education level variable with mother's employment variable which was reflected in a significant impact on children's screen viewing time. We believe that this study contributes to the available literature because it evaluates sleep and behavior quality by six factors of sleep disturbance and three behavioral indicators. Moreover, the tool used is in Arabic the native language of the recruited sample, was validated and found to produce reliable scores (0.71, 0.78). In addition, the questionnaire measured parents' perceptions and observations rather than child self-reporting and was reinforced by telephone interviews. Another feature of this study is the inclusion of very young age (18–36 months) among the recruited children. The scales used are appropriate to this age group. For the Children's Behavior Questionnaire (CBQ) the first form is for children aged 3–7 years (9). The second form is for children aged 18–36 months (10). The statistical analysis, the internal consistency and the factor analysis support the use of SDSC as an evaluation tool even at preschool age (range 3–6 years) (11). Finally applying multivariate regression analysis helped study the strength of association between the various technologic and demographic factors with sleep and behavior scores.

The study found that the ill effect of screen viewing or digital device use on behavior scores was dose dependent and was worse with 3–5 h of use. Owning a device, use of the tablet, and watching movies were associated with negative impact on behavior and sleep. Similarly, higher family income, lower educational level of the father, the longer working or studying hours for the father were associated with negative behavior scores. Regarding sleep, duration of ≥3–5 h was strongly associated with higher sleep disturbance scores. Television and smartphone devices and movies content had the worst impact on sleep quality. Unemployment or long hours of work/study for the mother had a significant ill effect on the child's sleep. The unemployment of the mother effect is probably related to disturbance of sleep/wake cycle of the whole family as unemployed mothers tend to stay up late at night and this might be reflected on the child sleep pattern. The finding of negative influence of technology or screen time viewing on sleep is in agreement with a previous systematic review of 67 studies that showed that screen time was associated with shortened duration and delayed onset of sleep in 90% of the studies (12). A recent study showed that children who use a smart phone or tablet within 90 min of sleep are twice as likely to have insufficient sleep (13, 14). The ill effect of television (TV) viewing on child language, behavior and sleep has gained some attention earlier on with emphasis on older children. Very few studies addressed children below 2 years of age, and the problem became more evident when TV programmers started targeting young children (7). Speculations about the reasons for the ill effect of technology or screen viewing ranged from mere interference with physical activity or replacement of sleep with smartphone or media screen viewing (7, 15–22) to a direct effect of screen viewing on melatonin secretion (23). Some studies focused on day-time sleepiness rather than measuring quality of sleep (24, 25).

Opposing studies have shown no relationship between technology or screen viewing and sleep quality (25, 26). Moreover, some studies use technology for diagnosis and treatment of behavioral and sleep disorders (27–29).

The use of media technologies negatively affects the psychosocial development of children (4), particularly their psychosocial development. The use of media has significantly changed in the past few years. However, researchers have not provided sufficient evidence for the impact of technology use on the health of children. The use of the available media technologies predominantly affects the health of pre-school children (5). In addition, the impact on their mental health, particularly self-concept and social competence, is of great concern. Therefore, parental monitoring is necessary to reduce the above-mentioned negative effects (3).

Appropriate parental monitoring enables children to engage in various media-related activities, with minimum negative effects on their psychosocial health. Children usually fail to perceive the negative health influence of heavy media usage. This can be attributed to the fact that they find great utility in this usage. Consequently, these children consider themselves more competent and receive a positive reward for such behavior (30). Nonetheless, this perception is usually false. Extensive media and video game usage has been associated with low social acceptance rather than any benefits (3).

The perception of parents toward the negative behavioral consequences of technology use by pre-school children is consistent with the existing scientific evidence, provided their parenting style does not purposefully focus on technology. Therefore, parents associate an increase in screen time with adverse behavioral effects in the youth. Furthermore, they argue that a failure to strategically use the screen time will certainly lead to internalizing and externalizing behavioral problems (31). According to families with pre-school children, increased screen time will not only affect early childhood but also middle childhood and adolescence, with the impact on adulthood remaining obscure.

Children are oblivious to the ill effect of mobile phones on their interpersonal relationships on daily basis. On the contrary, they tend to think that technology facilitates maintaining or managing friendships and other relationships (32).

Television, mobile phones, and tablets are the most frequently used devices in SA. Furthermore, they are frequently used by pre-school children and have been associated with increased behavioral problems (2). However, lack of awareness of parents of the influence of technology on children psychosocial development is the primary issue in SA. The parental impression about the effects of technology use on children and their behavior is rather neutral. However, mothers tend to be more concerned about this issue, compared to fathers.

In a study investigating the impact of television on the quality of sleep in preschool children, 43% of the SDSC of the children included in the study was abnormal. Children with a TV set inside their bedroom showed significantly higher scores in the “sleep terrors,” “nightmares,” “sleep talking,” and “tired when waking up” items of the SDSC (*P* = 0.02, 0.01, 0.02, and 0.01, respectively). The total hours of watching TV and the DIMS subscale score also have a significant correlation. Furthermore, watching TV after 20:00 was more frequent in the SDSC + group than in those with a normal SDSC score, 55 vs. 33%, respectively (*P* = 0.02). Evening TV viewers had significantly higher scores in the DIMS, SBD, and SDSC total score compared with those who watched TV earlier during the day (*P* = 0.02, 0.01, and 0.04, respectively) (4). The findings of this study are in conjunction with our own findings wherein the content of media the children is exposed to is significantly related to SBD. In our findings, children who most commonly watch movies have a higher SBD score than children who most commonly watched cartoons.

Insufficient sleep worsens or facilitates the emergence of emotional and behavioral control problems in children with pre-existent difficulties regulating affect (33). Proportion of media use in one study is significantly related to lower sleep efficacy at values of effortful control lower than or equal to −0.37 (34). In our study, effortful control is significantly correlated with the presence of self-owned electronic device by the child. In addition, effortful control and SBD had a significant correlation among other sleep disturbance scores. This is somehow in line with the findings in a study by Nathanson et al. (35) wherein there is a significant and negative relation between young children's tablet time and their EC, but only among children who received <10.61 h of sleep at night, which reflected about 40% of the children in their study. In addition to their results, a positive EC correlation can be detected in handheld game players only when children have more than 10.42 h of sleep. In our results, there is no significant difference in EC and other sleep disturbance parameters except for SBD with a weak positive correlation (r-0.18). It may be that despite the trends in higher duration of technology use by the children in our study, adequate sleep time is still being imposed, which is more than 10.61 h (35). In our study, there is more focus on sleep quality than sleep quantity.

## Conclusion

This is the first large scale study in SA to measure the impact of technology on behavior and sleep disturbance in pre-school children by using a validated Arabic version of the SDSC and CBQ. The findings of this study provide evidence for a dose-dependent negative impact of technology use on the sleep quality and behavioral pattern of children.

## Recommendation

Early intervention by admitting preschool children to nursery and kindergarten, censoring the duration and content of technology use, spending more time with family, and encouraging participation in motor activities is recommended to decrease the impact of technology on behavior and sleep disturbance (6).

## Limitation

We did not have a control group of children, which limited some of the generalization of the findings. Hence, this may not represent a cause and effect but just an association. We do not really know the baseline sleep and behavior ratings of children of similar ages within the population who were not exposed to technology. The possibility of response bias cannot be excluded as about half of respondents were in the mid to higher-income levels.

## Data Availability Statement

The raw data supporting the conclusions of this article will be made available by the authors, without undue reservation.

## Ethics Statement

The study received ethical approval from the ethical review board of Imam Abdulrahman Bin Faisal University. Consent was taken at the beginning of the interview and in writing at the beginning of the survey.

## Author Contributions

DA: first author, idea, and methodology. AAla: data collection and interviewer supervisor. MAlm: methodology. MZ: statistical analysis. NA: dissuction part. MAls: result part. FA: introduction and questionnaires. MAlm: result part and dissuction part. AAla: introduction and questionnaires. All authors contributed to the article and approved the submitted version.

## Conflict of Interest

The authors declare that the research was conducted in the absence of any commercial or financial relationships that could be construed as a potential conflict of interest.
