# Supplementary material for: Impact of Technology Use on Behavior and Sleep Scores in Preschool Children in Saudi Arabia
Source: Front Psychiatry. 2021 May 21;12:649095. doi: 10.3389/fpsyt.2021.649095 (PMC8175968; doi:10.3389/fpsyt.2021.649095)
Supplement: Supplementary file 1 [file Data_Sheet_1.docx]

Supplementary Material

**Supplementary table 1: Characteristics of the study participants**

|  | N | % |
| --- | --- | --- |
| **Age group** | | |
| 18 - 36 months | 71 | 24.7 |
| 3 - 5 years | 217 | 75.3 |
| **Child gender** | | |
| Male | 145 | 50.3 |
| Female | 143 | 49.7 |
| **Region** | | |
| Eastern | 180 | 62.5 |
| Central | 108 | 37.5 |
| **School** | | |
| Kindergarden | 65 | 22.6 |
| Nursery | 41 | 14.2 |
| Not going to school | 182 | 63.2 |
| **The number of the child between his/her siblings, mean (Standard deviation (SD))** | 2.6 (1.8) | |
| **Number of siblings in his/her family, mean (SD)** | 2.7 (1.9) | |
| **Marital status of parents** | | |
| Divorced | 25 | 8.6 |
| Married | 261 | 90.6 |
| Widowed | 2 | 0.7 |
| **Housing** | | |
| Owned | 144 | 50.0 |
| Rented | 144 | 50.0 |
| **Socioeconomic status** | | |
| <5,000SR | 36 | 12.5 |
| 5,000–10,000SR | 87 | 30.2 |
| 10,000–15,000SR | 80 | 27.8 |
| >15,000SR | 85 | 29.5 |
| **Highest educational attainment of the father** | | |
| Post-graduate | 43 | 14.9 |
| Bachelor | 129 | 44.8 |
| Intermediate | 68 | 23.6 |
| Diploma | 44 | 15.3 |
| Elementary school | 3 | 1.0 |
| No education | 1 | 0.3 |
| **Father was employed or studying** | | |
| Yes | 262 | 91.0 |
| No | 26 | 9.0 |
| **If the father was employed or studying, number of working or studying hours per week, mean (SD)** | 42.6(14) | |
| **Highest educational attainment of the mother** | | |
| Post-graduate | 25 | 8.7 |
| Bachelor | 177 | 61.5 |
| Intermediate | 60 | 20.8 |
| Diploma | 24 | 8.3 |
| Elementary school | 2 | 0.7 |
| **Mother was employed or studying** | | |
| Yes | 98 | 34.0 |
| No | 190 | 66.0 |
| **If the mother was employed or studying, the number of working or studying hours per week, mean (SD)** | 37.7 (10.1) | |

**Supplementary table 2: Technology use by the children**

|  | N | % |
| --- | --- | --- |
| **Type of technology used (multiple choices allowed)** | | |
| Television | 215 | 74.7 |
| Smart phone | 172 | 59.7 |
| Tablet | 98 | 34.0 |
| Video games | 25 | 8.7 |
| Laptop | 2 | 0.7 |
| **Most common device** | | |
| Smart phones | 122 | 42.3 |
| Television | 112 | 38.8 |
| Tablet | 39 | 13.5 |
| Video games | 15 | 5.2 |
| **Technology use per day** | | |
| ≤1 h | 76 | 26.4 |
| 2-3 h | 98 | 34.0 |
| 3-5 h | 66 | 22.9 |
| >5 h | 48 | 16.7 |
| **Children’s preferences during free time** | | |
| Family | 159 | 55.2 |
| Technology | 89 | 30.9 |
| Friends | 40 | 13.9 |
| **Type of content viewed** | | |
| Games | 103 | 35.8 |
| Videos | 67 | 23.3 |
| Education | 54 | 18.8 |
| Music | 51 | 17.7 |
| Cartoon | 4 | 1.4 |
| Both education and game | 3 | 1.0 |
| Family vlog | 3 | 1.0 |
| Blogs | 1 | 0.3 |
| All of them | 1 | 0.3 |
| None | 1 | 0.3 |
| **Use of technology** | | |
| Alone | 75 | 26.0 |
| With family | 213 | 74.0 |
| **Does your child have access to the internet at home?** | | |
| No | 103 | 35.8 |
| Yes | 185 | 64.2 |
| **Does your child own a device?** | | |
| Tablet | 57 | 19.8 |
| Smart phone | 31 | 10.8 |
| Video games | 13 | 4.5 |
| Does not have any | 187 | 64.9 |
| **Do you set watching or playing limits on technology use?** | | |
| No | 62 | 21.5 |
| Yes | 226 | 78.5 |
| **Do you supervise the child when he/she uses technology?** | | |
| No | 20 | 6.9 |
| Yes | 268 | 93.1 |
| **Most commonly viewed content** | | |
| Cartoons | 121 | 42.0 |
| Songs | 97 | 33.7 |
| Educational | 33 | 11.5 |
| Games | 37 | 12.8 |
| **Content (multiple answers allowed)** | | |
| Songs | 130 | 45.1 |
| Comedy | 85 | 29.5 |
| Educational | 83 | 28.8 |
| Action | 40 | 13.9 |
| Fantasy | 37 | 12.8 |
| Cartoons | 18 | 6.3 |
| Lifestyle | 17 | 5.9 |
| Games | 14 | 4.9 |
| Family vlog | 9 | 3.1 |
| Horror | 8 | 2.8 |
| Mystery | 5 | 1.7 |
| Learning English | 3 | 1.0 |
| Dancing | 3 | 1.0 |
| Roughness | 2 | 0.7 |
| Children vlog | 2 | 0.7 |
| Blogs | 1 | 0.3 |
| Pranks | 1 | 0.3 |
| Invention videos | 1 | 0.3 |
| Fortnight | 1 | 0.3 |
| Kids channel | 1 | 0.3 |
| Stories | 1 | 0.3 |

**Supplementary table 3: The association between the behavior and sleep disturbance scores with the most commonly used device**

|  | | **N** | **Mean** | **Standard Deviation** | **P-value*** | **Pairwise comparisons**** |
| --- | --- | --- | --- | --- | --- | --- |
|  |  |  |  |  |  |  |
| **DIMS** | Smart phone | 122 | 12.6 | 4.7 | 0.140 |  |
|  | Tablet | 39 | 11.0 | 3.3 |  |  |
|  | Television | 112 | 12.8 | 4.6 |  |  |
|  | Video games | 14 | 11.4 | 3.4 |  |  |
| **SBD** | Smart phone | 122 | 3.8 | 2.3 | 0.127 |  |
|  | Tablet | 39 | 3.2 | 0.7 |  |  |
|  | Television | 112 | 3.3 | 0.7 |  |  |
|  | Video games | 14 | 3.1 | 0.3 |  |  |
| **DA** | Smart phone | 122 | 3.9 | 1.8 | 0.845 |  |
|  | Tablet | 39 | 3.5 | 1.0 |  |  |
|  | Television | 112 | 3.8 | 1.5 |  |  |
|  | Video games | 14 | 3.6 | 0.7 |  |  |
| **SWTD** | Smart phone | 122 | 8.2 | 3.1 | 0.423* |  |
|  | Tablet | 39 | 7.7 | 1.8 |  |  |
|  | Television | 112 | 7.8 | 2.7 |  |  |
|  | Video games | 14 | 8.7 | 1.4 |  |  |
| **DOES** | Smart phone | 122 | 7.8 | 3.8 | **0.005** | Video games<television video games<smart phones |
|  | Tablet | 39 | 6.7 | 2.5 |  |  |
|  | Television | 112 | 7.1 | 2.9 |  |  |
|  | Video games | 14 | 5.4 | 1.2 |  |  |
| **SHY** | Smart phone | 122 | 2.8 | 1.9 | 0.335 |  |
|  | Tablet | 39 | 2.3 | 0.9 |  |  |
|  | Television | 112 | 2.7 | 1.8 |  |  |
|  | Video games | 14 | 2.4 | 1.3 |  |  |
| **TOTAL** | Smart phone | 122 | 39.1 | 12.1 | 0.112 |  |
|  | Tablet | 39 | 34.3 | 5.8 |  |  |
|  | Television | 112 | 37.5 | 8.5 |  |  |
|  | Video games | 14 | 34.6 | 5.3 |  |  |
| **Surgency** | Smart phone | 122 | 4.4 | 1.1 | 0.796* |  |
|  | Tablet | 39 | 4.5 | 0.8 |  |  |
|  | Television | 112 | 4.5 | 1.0 |  |  |
|  | Video games | 14 | 4.4 | 1.2 |  |  |
| **Negative Effect** | Smart phone | 122 | 3.8 | 1.0 | 0.726* |  |
|  | Tablet | 39 | 4.0 | 1.1 |  |  |
|  | Television | 112 | 4.0 | 1.0 |  |  |
|  | Video games | 14 | 3.9 | 0.7 |  |  |
| **Effortful Control** | Smart phone | 122 | 4.2 | 1.6 | **<0.001** | smart phones<television smart phones<tablet |
|  | Tablet | 39 | 5.2 | 1.7 |  |  |
|  | Television | 112 | 5.0 | 1.2 |  |  |
|  | Video games | 14 | 4.6 | 1.3 |  |  |

*One-way ANOVA was used for those comparisons; The Kruskal-Wallis test was used for other comparisons. Significant P-values are presented in bold.

**Bonferroni test was used for the pairwise comparisons.

**DIMS**= **D**isorders of **I**nitiating and **M**aintaining **S**leep, **SBD**= **S**leep-**B**reathing **D**isorders, **DA**= **D**isorders of **A**rousal, **SWTD**= **S**leep-**W**ake **T**ransition **D**isorders, **DOES**= **D**isorders **O**f **E**xcessive **S**omnolence, **SHY**= **S**leep **H**yperhydrosis

**Supplementary table 4: The association between the behavior and sleep disturbance scores with the duration of technology use**

|  |  | N | Mean | Standard Deviation | P-value* | Pairwise comparisons** |
| --- | --- | --- | --- | --- | --- | --- |
| DIMS | ≤1 h | 76 | 11.4 | 4.3 | 0.159 |  |
|  | 2-3 h | 98 | 12.8 | 4.9 |  |  |
|  | 3-5 h | 66 | 12.5 | 3.8 |  |  |
|  | >5 h | 48 | 13.0 | 4.5 |  |  |
| SBD | ≤1 h | 76 | 3.6 | 1.2 | 0.121 |  |
|  | 2-3 h | 98 | 3.2 | 0.7 |  |  |
|  | 3-5 h | 66 | 3.7 | 2.0 |  |  |
|  | >5 h | 48 | 3.8 | 2.5 |  |  |
| DA | ≤1 h | 76 | 3.8 | 1.6 | 0.440 |  |
|  | 2-3 h | 98 | 3.6 | 1.1 |  |  |
|  | 3-5 h | 66 | 4.0 | 1.4 |  |  |
|  | >5 h | 48 | 4.0 | 2.3 |  |  |
| SWTD | ≤1 h | 76 | 7.8 | 2.8 | 0.670 |  |
|  | 2-3 h | 98 | 8.0 | 2.7 |  |  |
|  | 3-5 h | 66 | 8.1 | 2.1 |  |  |
|  | >5 h | 48 | 8.4 | 3.5 |  |  |
| DOES | ≤1 h | 76 | 7.3 | 3.1 | 0.292 |  |
|  | 2-3 h | 98 | 7.2 | 3.3 |  |  |
|  | 3-5 h | 66 | 6.8 | 2.5 |  |  |
|  | >5 h | 48 | 8.0 | 4.0 |  |  |
| SHY | ≤1 h | 76 | 3.2 | 2.2 | **0.010** | 1h or less> 2 to 3 hs |
|  | 2-3 h | 98 | 2.4 | 1.2 |  |  |
|  | 3-5 h | 66 | 2.8 | 1.7 |  |  |
|  | >5 h | 48 | 2.4 | 1.5 |  |  |
| TOTAL | ≤1 h | 76 | 36.9 | 10.4 | 0.496 |  |
|  | 2-3 h | 98 | 37.2 | 8.8 |  |  |
|  | 3-5 h | 66 | 37.8 | 6.8 |  |  |
|  | >5 h | 48 | 39.5 | 14.1 |  |  |
| Surgency | ≤1 h | 76 | 4.6 | 1.0 | 0.728 |  |
|  | 2-3 h | 98 | 4.4 | 1.1 |  |  |
|  | 3-5 h | 66 | 4.5 | 1.0 |  |  |
|  | >5 h | 48 | 4.5 | 0.9 |  |  |
| Negative Effect | ≤1 h | 76 | 3.7 | 1.0 | **0.009** | 1h or less< 3 to 5 hs |
|  | 2-3 h | 98 | 4.0 | 1.0 |  |  |
|  | 3-5 h | 66 | 4.2 | 1.0 |  |  |
|  | >5 h | 48 | 3.8 | 0.9 |  |  |
| Effortful Control | ≤1 h | 76 | 4.8 | 1.5 | 0.851 |  |
|  | 2-3 h | 98 | 4.6 | 1.7 |  |  |
|  | 3-5 h | 66 | 4.7 | 1.4 |  |  |
|  | >5 h | 48 | 4.7 | 1.6 |  |  |

*One-way ANOVA was used for the comparisons; the significant P-values are presented in bold.

**Bonferroni test was used for the pairwise comparisons.

**DIMS**= **D**isorders of **I**nitiating and **M**aintaining **S**leep, **SBD**= **S**leep-**B**reathing **D**isorders, **DA**= **D**isorders of **A**rousal, **SWTD**= **S**leep-**W**ake **T**ransition **D**isorders, **DOES**= **D**isorders **O**f **E**xcessive **S**omnolence, **SHY**= **S**leep **H**yperhydrosis

**Supplementary table 5: The association between the behavior and sleep disturbance scores with the socioeconomic status**

|  | | **N** | **Mean** | **Standard Deviation** | **P-value*** | **Pairwise comparisons**** |
| --- | --- | --- | --- | --- | --- | --- |
|  |  |  |  |  |  |  |
| **DIMS** | <5,000SR | 36 | 13.1 | 6.1 | 0.503 |  |
|  | 5,000-10,000SR | 87 | 12.4 | 3.6 |  |  |
|  | 10,000-15,000SR | 80 | 11.8 | 4.3 |  |  |
|  | >15,000SR | 85 | 12.6 | 4.6 |  |  |
| **SBD** | <5,000SR | 36 | 3.5 | 1.0 | 0.665 |  |
|  | 5,000-10,000SR | 87 | 3.7 | 2.0 |  |  |
|  | 10,000-15,000SR | 80 | 3.5 | 1.8 |  |  |
|  | >15,000SR | 85 | 3.4 | 1.0 |  |  |
| **DA** | <5,000SR | 36 | 3.7 | 1.4 | 0.475 |  |
|  | 5,000-10,000SR | 87 | 4.0 | 2.0 |  |  |
|  | 10,000-15,000SR | 80 | 3.6 | 1.2 |  |  |
|  | >15,000SR | 85 | 3.8 | 1.5 |  |  |
| **SWTD** | <5,000SR | 36 | 7.8 | 2.9 | 0.098 |  |
|  | 5,000-10,000SR | 87 | 8.5 | 3.7 |  |  |
|  | 10,000-15,000SR | 80 | 7.5 | 1.7 |  |  |
|  | >15,000SR | 85 | 8.1 | 2.3 |  |  |
| **DOES** | <5,000SR | 36 | 7.2 | 3.3 | **0.018** | 5000-10000> 10000-15000 |
|  | 5,000-10,000SR | 87 | 8.1 | 3.9 |  |  |
|  | 10,000-15,000SR | 80 | 6.7 | 2.2 |  |  |
|  | >15,000SR | 85 | 6.9 | 3.2 |  |  |
| **SHY** | <5,000SR | 36 | 4.0 | 3.0 | **<0.001** | <5000 is higher than all others |
|  | 5,000-10,000SR | 87 | 2.7 | 1.6 |  |  |
|  | 10,000-15,000SR | 80 | 2.4 | 1.2 |  |  |
|  | >15,000SR | 85 | 2.4 | 1.3 |  |  |
| **TOTAL** | <5,000SR | 36 | 39.2 | 11.5 | 0.064 |  |
|  | 5,000-10,000SR | 87 | 39.4 | 11.9 |  |  |
|  | 10,000-15,000SR | 80 | 35.5 | 7.0 |  |  |
|  | >15,000SR | 85 | 37.2 | 9.0 |  |  |
| **Surgency** | <5,000SR | 36 | 4.5 | 1.2 | 0.145 |  |
|  | 5,000-10,000SR | 87 | 4.3 | 1.0 |  |  |
|  | 10,000-15,000SR | 80 | 4.6 | 0.9 |  |  |
|  | >15,000SR | 85 | 4.6 | 1.0 |  |  |
| **Negative Effect** | <5,000SR | 36 | 3.9 | 1.1 | 0.536 |  |
|  | 5,000-10,000SR | 87 | 3.9 | 1.1 |  |  |
|  | 10,000-15,000SR | 80 | 4.1 | 1.0 |  |  |
|  | >15,000SR | 85 | 3.8 | 1.0 |  |  |
| **Effortful Control** | <5,000SR | 36 | 4.4 | 1.7 | 0.243 |  |
|  | 5,000-10,000SR | 87 | 4.5 | 1.8 |  |  |
|  | 10,000-15,000SR | 80 | 4.9 | 1.4 |  |  |
|  | >15,000SR | 85 | 4.7 | 1.3 |  |  |

*One-way ANOVA was used for the comparisons; the significant P-values are presented in bold.

**Bonferroni test was used for the pairwise comparisons.

**DIMS**= **D**isorders of **I**nitiating and **M**aintaining **S**leep, **SBD**= **S**leep-**B**reathing **D**isorders, **DA**= **D**isorders of **A**rousal, **SWTD**= **S**leep-**W**ake **T**ransition **D**isorders, **DOES**= **D**isorders **O**f **E**xcessive **S**omnolence, **SHY**= **S**leep **H**yperhydrosis

**Supplementary table 6: The association between the behavior and sleep disturbance scores with the most commonly used content**

|  | | **N** | **Mean** | **Standard Deviation** | **P-value*** | **Pairwise comparisons**** |
| --- | --- | --- | --- | --- | --- | --- |
| **DIMS** | Movies | 121 | 11.8 | 4.4 | 0.285 |  |
|  | Songs | 97 | 12.6 | 4.3 |  |  |
|  | Educational | 33 | 13.2 | 5.5 |  |  |
|  | Games/cartoons | 37 | 12.9 | 4.3 |  |  |
| **SBD** | Movies | 121 | 3.8 | 2.3 | **0.024** | Movies > Games/cartoons |
|  | Songs | 97 | 3.3 | 0.8 |  |  |
|  | Educational | 33 | 3.4 | 0.9 |  |  |
|  | Games/cartoons | 37 | 3.0 | 0.2 |  |  |
| **DA** | Movies | 121 | 3.8 | 1.5 | 0.484 |  |
|  | Songs | 97 | 3.6 | 1.1 |  |  |
|  | Educational | 33 | 3.9 | 2.4 |  |  |
|  | Games/cartoons | 37 | 4.1 | 1.8 |  |  |
| **SWTD** | Movies | 121 | 8.0 | 2.4 | 0.352 |  |
|  | Songs | 97 | 7.8 | 2.7 |  |  |
|  | Educational | 33 | 8.8 | 4.5 |  |  |
|  | Games/cartoons | 37 | 7.9 | 2.0 |  |  |
| **DOES** | Movies | 121 | 6.9 | 3.0 | 0.401 |  |
|  | Songs | 97 | 7.6 | 3.2 |  |  |
|  | Educational | 33 | 7.2 | 4.1 |  |  |
|  | Games/cartoons | 37 | 7.6 | 3.1 |  |  |
| **SHY** | Movies | 121 | 2.7 | 1.5 | **0.007** | Educational>Games/cartoons |
|  | Songs | 97 | 2.6 | 1.5 |  |  |
|  | Educational | 33 | 3.5 | 2.9 |  |  |
|  | Games/cartoons | 37 | 2.2 | 0.7 |  |  |
| **TOTAL** | Movies | 121 | 37.0 | 9.4 | 0.471 |  |
|  | Songs | 97 | 37.6 | 9.4 |  |  |
|  | Educational | 33 | 40.1 | 13.8 |  |  |
|  | Games/cartoons | 37 | 37.6 | 9.1 |  |  |
| **Surgency** | Movies | 121 | 4.4 | 1.0 | 0.444 |  |
|  | Songs | 97 | 4.5 | 1.1 |  |  |
|  | Educational | 33 | 4.4 | 0.9 |  |  |
|  | Games/cartoons | 37 | 4.7 | 0.9 |  |  |
| **Negative Effect** | Movies | 121 | 4.0 | 1.1 | 0.091 |  |
|  | Songs | 97 | 3.7 | 0.9 |  |  |
|  | Educational | 33 | 3.9 | 1.0 |  |  |
|  | Games/cartoons | 37 | 4.2 | 1.2 |  |  |
| **Effortful Control** | Movies | 121 | 4.5 | 1.6 | **0.023** | Movies<Games/cartoons |
|  | Songs | 97 | 4.5 | 1.5 |  |  |
|  | Educational | 33 | 5.0 | 1.4 |  |  |
|  | Games/cartoons | 37 | 5.3 | 1.2 |  |  |

*One-way ANOVA was used for the comparisons; the significant P-values are presented in bold.

**Bonferroni test was used for the pairwise comparisons.

**DIMS**= **D**isorders of **I**nitiating and **M**aintaining **S**leep, **SBD**= **S**leep-**B**reathing **D**isorders, **DA**= **D**isorders of **A**rousal, **SWTD**= **S**leep-**W**ake **T**ransition **D**isorders, **DOES**= **D**isorders **O**f **E**xcessive **S**omnolence, **SHY**= **S**leep **H**yperhydrosis
